# Association Between Hepatitis C Virus Infection and SYNTAX Score in Patients with ST-Segment Elevation Myocardial Infarction: A Propensity Score-Matched Analysis

**DOI:** 10.3390/jcm15114180

**Published:** 2026-05-28

**Authors:** Ismail Balaban, Seda Tanyeri Uzel, Elif Caglayan, Dogancan Ceneli, Halit Eminoglu, Barkin Kultursay, Mustafa Ferhat Keten, Kadir Biyikli

**Affiliations:** 1 Department of Cardiology, Hamidiye School of Medicine, Kosuyolu Heart, Education and Research Institute, University of Health Sciences, Istanbul 34865, Turkey; sedatanyeri@hotmail.com (S.T.U.); dogancanceneli@yahoo.com (D.C.); eminogluhalit@gmail.com (H.E.); ferhat_keten@hotmail.com (M.F.K.); kadirbiyikli93@hotmail.com (K.B.); 2 Department of Microbiology, Hamidiye School of Medicine, Kosuyolu Heart, Education and Research Institute, University of Health Sciences, Istanbul 34865, Turkey; elifcaglayan@hotmail.com; 3 Department of Cardiology, Tunceli State Hospital, Tunceli 62000, Turkey; bkultursay@gmail.com

**Keywords:** coronary artery disease, hepatitis C, propensity score matching, ST-segment elevation myocardial infarction, SYNTAX score

## Abstract

**Background:** Chronic hepatitis C virus (HCV) infection is increasingly recognized as a systemic inflammatory condition associated with accelerated atherosclerosis and adverse cardiovascular outcomes. However, its relationship with coronary anatomical complexity in patients presenting with ST-segment elevation myocardial infarction (STEMI) remains insufficiently defined. **Aims:** This study aimed to evaluate the association between chronic HCV infection and coronary artery disease complexity assessed by the SYNTAX score in STEMI patients undergoing primary percutaneous coronary intervention (pPCI) and to investigate its relationship with in-hospital mortality. **Methods:** In this retrospective single-center cohort study, 1647 STEMI patients treated with pPCI between January 2021 and December 2025 were analyzed; 106 (6.4%) were HCV-positive. Propensity score matching based on baseline demographic and cardiovascular risk factors yielded 105 matched pairs. The primary endpoint was the SYNTAX score, while the secondary endpoint was in-hospital all-cause mortality. **Results:** HCV-positive patients demonstrated significantly higher SYNTAX scores than HCV-negative patients before (19.5 ± 8.5 vs. 15.8 ± 9.6; *p* < 0.001) and after propensity score matching (19.4 ± 8.5 vs. 15.6 ± 9.2; *p* = 0.002). Coronary artery bypass grafting referral was more frequent among HCV-positive patients both before (11.3% vs. 5.3%; *p* = 0.010) and after matching (11.4% vs. 2.9%; *p* = 0.016). Notably, HCV-positive patients exhibited higher coronary anatomical complexity despite lower total and LDL cholesterol levels. In multivariable analyses, HCV positivity remained independently associated with higher SYNTAX scores in both unmatched and matched cohorts. In-hospital mortality rates were comparable between groups, and HCV positivity was not independently associated with mortality. **Conclusions:** Chronic HCV infection was independently associated with increased coronary anatomical complexity in STEMI patients undergoing pPCI, suggesting a relationship with a more diffuse and structurally complex atherosclerotic phenotype rather than short-term in-hospital outcomes. These findings support the concept of HCV infection as a non-traditional cardiovascular risk factor associated with adverse coronary vascular remodeling.

## 1. Introduction

Chronic hepatitis C virus (HCV) infection is increasingly recognized as a systemic inflammatory condition with clinically relevant extrahepatic manifestations extending beyond the liver. In addition to hepatic injury, chronic HCV infection has been consistently associated with metabolic disturbances, immune dysregulation, and increased cardiovascular risk [1]. Persistent immune activation, oxidative stress, endothelial dysfunction, and chronic low-grade inflammation are considered central mechanisms through which HCV may contribute to accelerated atherosclerotic disease progression [2,3].

Emerging evidence suggests that chronic HCV infection contributes to cardiovascular disease through mechanisms extending beyond traditional cardiovascular risk factors. These mechanisms include endothelial dysfunction, oxidative stress, immune-mediated vascular injury, insulin resistance, and a prothrombotic inflammatory milieu [3,4,5]. A meta-analysis by Wen et al. demonstrated that HCV infection was associated with a significantly increased risk of coronary artery disease, supporting the concept of HCV as a non-traditional cardiovascular risk factor [6]. Furthermore, recent contemporary studies have continued to demonstrate associations between HCV infection and both subclinical and clinically manifest atherosclerotic cardiovascular disease [7].

Endothelial dysfunction represents a key pathophysiological link between chronic inflammation and atherosclerosis. Experimental and clinical studies have shown that HCV infection is associated with impaired endothelial function, increased oxidative stress, and reduced nitric oxide bioavailability, thereby promoting vascular injury and plaque formation [2,4]. In parallel, HCV-related metabolic abnormalities—including insulin resistance, hepatic steatosis, and systemic inflammatory activation—may further accelerate atherogenesis, even in patients without markedly adverse conventional lipid profiles [5,8].

Consistent with these mechanisms, multiple observational studies have demonstrated associations between HCV infection and both subclinical and clinical atherosclerosis. Prospective and cross-sectional studies have reported increased carotid intima–media thickness and higher prevalence of carotid plaque among HCV-infected individuals compared with uninfected controls [5,9]. Moreover, large population-based cohorts and meta-analyses have shown significantly increased risks of coronary artery disease, acute coronary syndromes, and cardiovascular mortality in patients with chronic HCV infection [8,10,11,12,13].

Despite accumulating evidence linking HCV infection with cardiovascular disease, the relationship between chronic HCV infection and the anatomical burden and complexity of coronary artery disease remains incompletely defined. Several angiographic and imaging-based studies have suggested that HCV-positive patients may exhibit more severe coronary atherosclerosis, increased vascular calcification, and multivessel disease, whereas other investigations have reported inconsistent findings, particularly in acute coronary syndrome populations [14,15,16,17]. Recently, Wang et al. demonstrated that HCV infection was independently associated with coronary and thoracic aortic atherosclerosis, especially in the presence of metabolic syndrome and advanced liver fibrosis [18]. Importantly, much of the existing literature has focused primarily on disease presence or cardiovascular event rates rather than on the overall anatomical complexity of coronary artery disease, which may better reflect the cumulative vascular effects of chronic inflammatory exposure.

The SYNTAX score provides a comprehensive angiographic assessment of coronary artery disease by integrating lesion number, anatomical location, and morphological complexity [19]. As such, it offers a robust measure of overall coronary atherosclerotic burden and has important implications for revascularization strategy and long-term prognosis. However, data examining the association between chronic HCV infection and SYNTAX-defined coronary complexity remain limited.

Furthermore, although chronic HCV infection has been associated with an increased long-term risk of acute coronary syndromes [20], its role in shaping the underlying coronary anatomy in patients presenting with ST-segment elevation myocardial infarction (STEMI) has not been adequately explored. STEMI represents an acute thrombotic event superimposed on underlying coronary atherosclerosis, and the extent of anatomical disease complexity strongly influences procedural strategy, completeness of revascularization, and long-term outcomes. Whether chronic HCV infection predisposes STEMI patients to a more diffuse or structurally complex coronary artery disease phenotype therefore remains an important unresolved question.

In this context, the present study aimed to evaluate the association between chronic HCV infection and coronary artery disease complexity, as assessed by the SYNTAX score, in patients presenting with ST-segment elevation myocardial infarction undergoing primary percutaneous coronary intervention.

## 2. Methods

### 2.1. Study Design

This study was designed as a retrospective, observational, single-center cohort study. The study was conducted in accordance with the principles of the Declaration of Helsinki and received approval from the Institutional Review Board of Kartal Koşuyolu Training and Research Hospital. Due to the retrospective nature of the study, the requirement for written informed consent was waived.

### 2.2. Patient Selection

**Figure 1:**
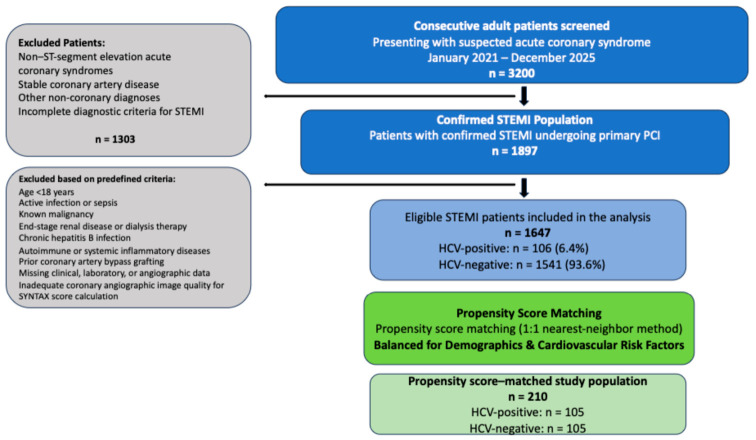
Study flow diagram and patient selection process.

Consecutive adult patients presenting with a diagnosis of ST-segment elevation myocardial infarction (STEMI) and undergoing primary percutaneous coronary intervention (pPCI) between January 2021 and December 2025 were screened for inclusion in this study (Figure 1). All eligible patients were identified retrospectively through the electronic hospital database.

Flow diagram illustrating patient selection and study design. A total of 3200 consecutive adult patients presenting with suspected acute coronary syndrome were screened. After exclusion of patients without ST-segment elevation myocardial infarction (STEMI), non-coronary diagnoses, and those meeting predefined exclusion criteria, 1647 patients with confirmed STEMI undergoing primary percutaneous coronary intervention were included in the analysis. Among these, 106 patients were positive for chronic hepatitis C virus (HCV) infection. Propensity score matching was subsequently performed using a 1:1 nearest-neighbor approach, yielding a final matched cohort of 210 patients (105 HCV-positive and 105 HCV-negative) with balanced baseline characteristics.

The diagnosis of STEMI was established according to contemporary guideline criteria, based on the presence of typical ischemic chest pain lasting at least 20 min, ST-segment elevation of ≥1 mm in contiguous electrocardiographic leads (with age- and sex-specific thresholds applied for leads V2–V3), and/or the presence of new-onset left bundle branch block [21,22]. All patients underwent emergency coronary angiography followed by pPCI within the guideline-recommended time window after symptom onset.

Only patients with a confirmed diagnosis of STEMI who were treated with pPCI were included in the study. Patients presenting with non–ST-segment elevation acute coronary syndromes or stable coronary artery disease were excluded. Additional exclusion criteria included age < 18 years, the presence of active infection or sepsis, known malignancy, end-stage renal disease or ongoing dialysis therapy, known chronic hepatitis B infection, autoimmune or other systemic inflammatory diseases, prior coronary artery bypass grafting, and incomplete clinical, laboratory, or angiographic data. Furthermore, patients without coronary angiographic images of sufficient quality for accurate SYNTAX score calculation were not included in the final analysis.

### 2.3. Definition of HCV Infection

Chronic hepatitis C virus infection was defined by the presence of anti-HCV antibody positivity together with detectable serum HCV RNA. Chronic HCV infection was considered in patients with persistent HCV RNA positivity for at least six months following acute infection [23]. HCV status was verified using hospital laboratory databases and infectious disease medical records.

### 2.4. Clinical, Demographic, and Laboratory Data

Demographic characteristics (age, sex), cardiovascular risk factors (hypertension, diabetes mellitus, smoking status), admission vital signs, laboratory parameters, and treatment-related data were retrospectively obtained from electronic medical records.

Laboratory parameters were measured using blood samples obtained at admission or within the first 24 h of hospitalization. Left ventricular ejection fraction was assessed within the first 24 h of admission by transthoracic echocardiography using the biplane Simpson method.

### 2.5. Coronary Angiography and SYNTAX Score Assessment

All patients underwent emergency coronary angiography via either femoral or radial access. The extent and anatomical complexity of coronary artery disease were assessed using the SYNTAX score [19,24].

For SYNTAX score calculation:

- All coronary lesions causing ≥50% diameter stenosis in vessels ≥1.5 mm in diameter were included.
- Lesion number, anatomical location, presence of total occlusion, bifurcation or trifurcation involvement, calcification, vessel tortuosity, thrombus presence, and diffuse disease were evaluated.

SYNTAX scores were calculated independently by at least two experienced interventional cardiologists who were blinded to HCV status, clinical data, and patient outcomes. In cases of disagreement, a third interventional cardiologist reviewed the angiograms to determine the final SYNTAX score.

Patients with anatomically complex multivessel coronary artery disease deemed unsuitable for complete percutaneous revascularization underwent multidisciplinary Heart Team evaluation for staged surgical revascularization after culprit-lesion PCI and initial clinical stabilization.

### 2.6. Study Endpoints

The primary endpoint of the study was the assessment of coronary artery disease anatomical complexity, as quantified by the SYNTAX score. The secondary endpoint was in-hospital all-cause mortality.

### 2.7. Statistical Analysis

Continuous variables are presented as mean ± standard deviation. Categorical variables are expressed as counts and percentages. Comparisons between hepatitis C virus (HCV)-positive and HCV-negative patients were performed using Student’s *t*-test or the Mann–Whitney U test for continuous variables, and the chi-square test or Fisher’s exact test for categorical variables, as appropriate.

To minimize potential confounding related to HCV positivity and imbalance in baseline characteristics between groups, propensity score matching (PSM) was applied. The propensity score, defined as the conditional probability of HCV positivity given observed covariates, was estimated using a multivariable logistic regression model including age, sex, body mass index, hypertension, diabetes mellitus, and smoking status. HCV-positive patients were matched 1:1 with HCV-negative patients using the nearest-neighbor matching method without replacement. Covariate balance before and after matching was assessed using absolute treatment–covariate correlations and visually evaluated with Love plots (Supplementary Figure S1). A marked reduction in imbalance across all covariates after matching was considered indicative of adequate balance.

Covariate balance (Love) plot displaying absolute treatment–covariate correlations for variables included in the propensity score model. Red squares represent unmatched samples, and blue triangles represent matched samples. The reduction in imbalance after matching indicates improved covariate balance between HCV-positive and HCV-negative groups.

Linear regression analyses were performed to identify factors associated with coronary artery disease anatomical complexity, quantified by the SYNTAX score. Variables demonstrating an association at *p* < 0.10 in univariable analyses or considered clinically relevant were entered into multivariable regression models. Regression analyses were conducted in both the unmatched cohort and the propensity score-matched cohort to assess the robustness and consistency of the findings.

In multivariable regression analyses, continuous variables were entered as continuous predictors, whereas clinically relevant categorical variables (e.g., HCV positivity, diabetes mellitus, hypertension, and smoking status) were included as dichotomous covariates. Multicollinearity was assessed using variance inflation factor analysis.

For the evaluation of in-hospital all-cause mortality, logistic regression analyses were performed. Univariable analyses were followed by multivariable models adjusting for clinically relevant covariates. Results are reported as odds ratios (ORs) with corresponding 95% confidence intervals (CIs).

All statistical analyses were conducted in both the unmatched population and the propensity score-matched cohort. Statistical analyses were performed using [SPSS 28.0 and R 4.4.1] software, and a two-sided *p* value < 0.05 was considered statistically significant.

## 3. Results

### 3.1. Study Population

During the study period, 3200 consecutive adult patients presenting with suspected acute coronary syndrome were screened. After exclusion of patients without ST-segment elevation myocardial infarction (STEMI), those with non-coronary diagnoses, and patients meeting predefined exclusion criteria, a total of 1647 patients with confirmed STEMI who underwent primary percutaneous coronary intervention (pPCI) were included in the final analysis. Among these patients, 106 (6.4%) were positive for chronic hepatitis C virus (HCV) infection, while 1541 (93.6%) were HCV negative. The patient selection process is summarized in Figure 1.

To minimize baseline differences between groups, propensity score matching was performed. Following 1:1 nearest-neighbor matching, 105 HCV-positive patients were successfully matched with 105 HCV-negative patients with comparable baseline demographic and clinical characteristics. The resulting propensity score-matched cohort (n = 210) constituted the population for subsequent adjusted analyses (Figure 1).

### 3.2. Baseline Clinical Characteristics

Baseline demographic, clinical, and laboratory characteristics of the study population before and after propensity score matching are summarized in Table 1.

Before matching, atrial fibrillation was more prevalent among HCV-positive patients compared with HCV-negative patients (14.2% vs. 4.7%; *p* < 0.001). Body mass index was lower in the HCV-positive group (27.1 ± 3.7 vs. 28.2 ± 4.7 kg/m^2^; *p* = 0.029), and serum albumin levels were also lower (3.7 ± 0.5 vs. 3.8 ± 0.4 g/dL; *p* = 0.022). In addition, C-reactive protein levels were significantly higher in HCV-positive patients (4.0 ± 3.0 vs. 2.7 ± 3.8 mg/L; *p* < 0.001). No significant differences were observed between groups with respect to age, sex distribution, hypertension, diabetes mellitus, smoking status, admission blood pressure, heart rate, or left ventricular ejection fraction prior to matching. Although prior statin and aspirin use were numerically lower among HCV-positive patients, these differences did not reach statistical significance before or after propensity score matching (Table 1).

After propensity score matching, baseline characteristics included in the matching process—namely age, sex, body mass index, hypertension, diabetes mellitus, and smoking status—were well balanced between HCV-positive and HCV-negative patients (Table 1). Covariate balance was assessed using absolute treatment–covariate correlations and demonstrated a marked reduction in imbalance after matching, as illustrated in Supplementary Figure S1. Variables not included in the propensity score model, such as atrial fibrillation and selected laboratory parameters, were evaluated descriptively and showed residual differences consistent with the underlying clinical profile of HCV infection.

### 3.3. Laboratory Findings

Laboratory parameters in the propensity score-matched cohort are summarized in Table 1. Platelet counts were significantly lower in HCV-positive patients compared with HCV-negative patients (230.8 ± 75.1 ×10^3^/L vs. 251.2 ± 73.5 ×10^3^/L; *p* = 0.048). Serum albumin levels also remained lower in the HCV-positive group after matching (3.7 ± 0.5 vs. 3.8 ± 0.3 g/dL; *p* = 0.038).

Although C-reactive protein levels tended to be higher among HCV-positive patients, this difference did not reach statistical significance following matching (*p* = 0.071). No significant differences were observed between the matched groups with respect to liver function tests, uric acid levels, total white blood cell counts, or differential leukocyte counts (Table 1).

Lipid profile parameters differed significantly between groups. Before propensity score matching, HCV-positive patients had lower total cholesterol levels (179.4 ± 54.4 vs. 198.1 ± 47.4 mg/dL; *p* < 0.001) and lower LDL cholesterol levels (98.9 ± 45.3 vs. 116.2 ± 38.6 mg/dL; *p* < 0.001) compared with HCV-negative patients. Similar differences persisted after matching, with significantly lower total cholesterol (179.8 ± 54.5 vs. 194.3 ± 37.3 mg/dL; *p* = 0.028) and LDL cholesterol levels (99.2 ± 45.4 vs. 122.3 ± 31.4 mg/dL; *p* < 0.001) in the HCV-positive group. HDL cholesterol and triglyceride levels were comparable between groups (Table 1).

### 3.4. Coronary Angiographic Findings and SYNTAX Score

Coronary angiographic characteristics and SYNTAX score distributions are presented in Table 1. Before propensity score matching, HCV-positive patients demonstrated greater coronary artery disease complexity, with a higher mean SYNTAX score compared with HCV-negative patients (19.5 ± 8.5 vs. 15.8 ± 9.6; *p* < 0.001). This difference persisted after matching. In the propensity score-matched cohort, SYNTAX scores remained significantly higher in HCV-positive patients than in HCV-negative patients (19.4 ± 8.5 vs. 15.6 ± 9.2; *p* = 0.002) (Table 1).

With respect to revascularization strategy following index coronary angiography, referral for surgical revascularization (coronary artery bypass grafting) was more frequent among HCV-positive patients both before matching (11.3% vs. 5.3%; *p* = 0.010) and after matching (11.4% vs. 2.9%; *p* = 0.016) (Table 1).

### 3.5. Determinants of SYNTAX Score

**Figure 2:**
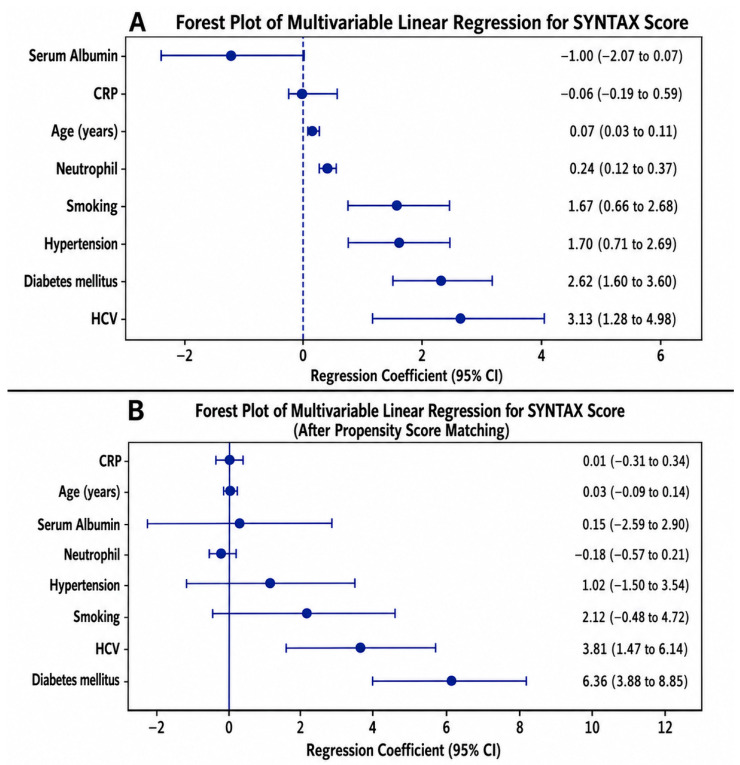
Multivariable linear regression analyses for SYNTAX score before and after propensity score matching: (**A**) Forest plot of multivariable linear regression in the unmatched cohort. Point estimates (β) and 95% confidence intervals (CIs) are shown for each covariate, with the dashed vertical line indicating β = 0. (**B**) Forest plot of the fully adjusted multivariable linear regression model in the propensity score-matched cohort, demonstrating independent determinants of SYNTAX score after balancing baseline covariates. Abbreviations: CI, confidence interval; CRP, C-reactive protein; HCV, hepatitis C virus.

The results of multivariable linear regression analyses evaluating factors associated with the SYNTAX score are shown in Table 2, Figure 2A (unmatched cohort), and Table 3, Figure 2B (propensity score-matched cohort).

In the unmatched cohort (Figure 2A), age, neutrophil count, diabetes mellitus, hypertension, smoking status, and HCV positivity were independently associated with higher SYNTAX scores. Serum albumin demonstrated an inverse association with coronary anatomical complexity. In this model, HCV positivity was associated with a significant increase in SYNTAX score (β = 3.13; 95% CI: 1.28–4.98), while diabetes mellitus also showed a strong positive association (β = 2.62; 95% CI: 1.60–3.60) (Table 2).

After propensity score matching (Figure 2B), the pattern of associations changed substantially. In the fully adjusted matched model, HCV positivity remained a robust and independent determinant of the SYNTAX score, corresponding to an approximately 3.8-point increase (β = 3.81; 95% CI: 1.47–6.14). Diabetes mellitus emerged as the strongest predictor of coronary anatomical complexity (β = 6.36; 95% CI: 3.88–8.85). In contrast, age, hypertension, smoking status, neutrophil count, inflammatory markers, and serum albumin were no longer statistically significant after matching (Table 3).

### 3.6. Variable Importance for High SYNTAX Score Prediction

**Figure 3:**
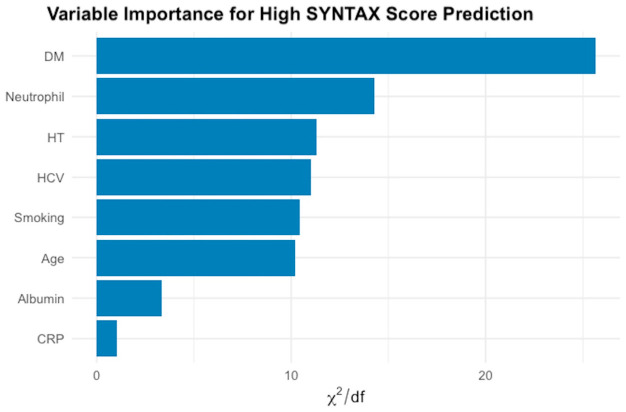
Variable importance of predictors for a high SYNTAX score. Abbreviations: CRP, C-reactive protein; DM, diabetes mellitus; HCV, hepatitis C virus; HT, hypertension.

The relative contribution of individual predictors to the presence of a high SYNTAX score is illustrated in Figure 3.

Variable importance plot demonstrating the relative contribution of clinical and laboratory variables to the prediction of a high SYNTAX score. Importance was quantified using the χ^2^/df metric derived from the regression model. Higher values indicate a greater relative contribution of the corresponding variable to SYNTAX score prediction.

Diabetes mellitus ranked as the most influential predictor, followed by neutrophil count and hypertension. Notably, HCV positivity ranked among the leading contributing variables, underscoring its substantial association with coronary anatomical complexity beyond traditional cardiovascular risk factors. Smoking status and age demonstrated moderate importance, whereas serum albumin and C-reactive protein showed comparatively lower contributions.

### 3.7. Nomogram for Individualized Prediction of SYNTAX Score

**Figure 4:**
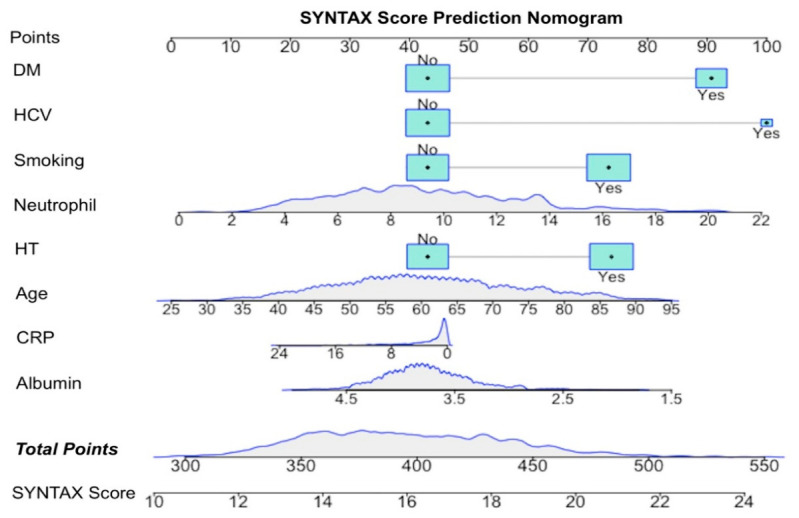
Nomogram for individualized prediction of SYNTAX score. Nomogram derived from the propensity score-matched multivariable regression model, illustrating the weighted contribution of each predictor to the estimated SYNTAX score. Abbreviations: CRP, C-reactive protein; DM, diabetes mellitus; HCV, hepatitis C virus; HT, hypertension.

Based on the propensity score-matched multivariable regression model, a nomogram was constructed to facilitate individualized estimation of the SYNTAX score (Figure 4). Each predictor was assigned a point value proportional to its regression coefficient, and the sum of points corresponds to the predicted SYNTAX score.

An illustrative example is shown in Supplementary Figure S2. For example, for a patient aged approximately 60 years who is HCV-positive but has no diabetes mellitus, no hypertension, and no active smoking, with relatively low CRP, preserved serum albumin, and a low neutrophil count, the total nomogram score is approximately 370 points, corresponding to an estimated SYNTAX score of ~14.6. This example demonstrates how HCV positivity may contribute to higher predicted coronary anatomical complexity even in the absence of several traditional risk factors.

### 3.8. In-Hospital Clinical Outcomes

In-hospital clinical outcomes are summarized in Table 4. In-hospital mortality rates were comparable between HCV-positive and HCV-negative patients, both before propensity score matching (10.4% vs. 10.2%; *p* = 0.950) and after matching (10.5% vs. 8.6%; *p* = 0.638).

In multivariable logistic regression analysis, age (OR: 1.055; 95% CI: 1.041–1.070; *p* < 0.001) and diabetes mellitus (OR: 1.964; 95% CI: 1.386–2.785; *p* < 0.001) were identified as independent predictors of in-hospital mortality. HCV positivity was not independently associated with in-hospital mortality (OR: 0.998; 95% CI: 0.513–1.940; *p* = 0.978) (Table 4).

## 4. Discussion

This study suggests that chronic hepatitis C virus (HCV) infection is associated with greater coronary anatomical complexity in patients presenting with ST-segment elevation myocardial infarction (STEMI). Rather than being interpreted as a direct trigger of acute coronary events, HCV infection may be viewed as a clinical correlate of the underlying structural substrate of coronary artery disease. This finding aligns with the growing body of evidence characterizing HCV as a systemic inflammatory condition with extrahepatic cardiovascular manifestations, potentially influencing long-term vascular remodeling beyond hepatic involvement [1].

Most prior investigations have focused on the association between HCV infection and the incidence of coronary events or cardiovascular mortality, yielding heterogeneous results [8,10,11,13]. While event-based analyses provide important prognostic insights, they offer limited information regarding the underlying coronary architecture. In contrast, coronary anatomical complexity reflects the cumulative burden of chronic pathological processes and has direct implications for revascularization strategy and long-term prognosis.

Angiographic studies evaluating coronary disease burden in HCV-infected patients have produced conflicting findings. Several reports have described more extensive or multivessel coronary artery disease among HCV-positive individuals [14,15,16], whereas others have found no significant differences compared with HCV-negative patients [17]. These discrepancies likely stem from differences in study populations, angiographic assessment methods, and the lack of standardized tools for capturing global coronary complexity.

The SYNTAX score integrates lesion number, anatomical location, and morphological characteristics, providing a comprehensive assessment of coronary artery disease burden. The observed association between HCV infection and higher SYNTAX scores in the present study suggests that chronic HCV infection may be associated with a more diffuse and structurally complex atherosclerotic phenotype, rather than isolated focal stenoses.

Several pathophysiological mechanisms may explain this association. Chronic HCV infection is characterized by persistent systemic inflammation, oxidative stress, endothelial dysfunction, and immune-mediated vascular injury, all of which contribute to atherogenesis and vascular remodeling [2,3,8]. Clinical studies have demonstrated impaired endothelial function and increased subclinical atherosclerosis in HCV-infected individuals, supporting the concept of early and sustained vascular injury in this population [4,9]. In addition, HCV-related metabolic disturbances, including insulin resistance and hepatic steatosis, may further accelerate atherosclerotic progression even in the absence of markedly adverse conventional lipid profiles [3,5].

One of the notable findings of the present study was that HCV-positive patients demonstrated significantly higher SYNTAX scores despite lower total and LDL cholesterol levels. This observation suggests that HCV-related atherosclerosis may not be fully explained by conventional lipid-mediated mechanisms. Instead, persistent inflammatory and vascular alterations associated with chronic HCV infection may contribute substantially to coronary anatomical complexity despite relatively favorable lipid profiles [7,25].

In the acute STEMI setting, coronary thrombosis develops on a background of pre-existing coronary atherosclerosis, and the extent of anatomical disease complexity remains an important determinant of procedural strategy and long-term prognosis. In this context, HCV infection may function primarily as a marker of advanced underlying coronary disease rather than as a determinant of short-term in-hospital clinical outcomes.

Although HCV-positive patients exhibited a higher prevalence of atrial fibrillation and a more pronounced inflammatory profile before matching, these differences did not translate into worse short-term clinical outcomes. In-hospital mortality rates were comparable between groups both before and after propensity score matching, and HCV positivity was not an independent predictor of mortality in multivariable analysis. These findings further support the interpretation that chronic HCV infection predominantly influences the underlying coronary atherosclerotic substrate rather than acute in-hospital clinical events.

The absence of a significant difference in in-hospital mortality between groups is consistent with several previous studies suggesting that the cardiovascular impact of chronic HCV infection may manifest predominantly over longer follow-up periods rather than during the acute hospitalization phase [8,13]. This interpretation is further supported by emerging evidence demonstrating that successful HCV eradication with direct-acting antiviral (DAA) therapy may improve endothelial function, reduce systemic inflammation, and decrease long-term cardiovascular risk [26,27,28].

Recent evidence also suggests that lower lipid levels in HCV-positive patients do not necessarily indicate lower cardiovascular risk, as chronic HCV infection may alter lipid metabolism while simultaneously promoting atherosclerotic processes through non-traditional mechanisms [29]. Therefore, conventional lipid measurements alone may underestimate the true vascular risk profile in patients with chronic HCV infection.

Although prior statin use was numerically lower in the HCV-positive group, the difference did not reach statistical significance. Previous studies have suggested that statins remain underutilized in patients with chronic HCV infection because of concerns regarding hepatotoxicity, despite growing evidence supporting their safety in compensated chronic liver disease [30,31]. In addition to cardiovascular protection, statin therapy has also been associated with reduced fibrosis progression and lower hepatocellular carcinoma risk in patients with chronic liver disease and HCV infection [31,32].

From a clinical perspective, recognizing chronic HCV infection as a non-traditional correlate of coronary disease complexity may improve cardiovascular risk stratification in patients presenting with STEMI. Awareness of HCV status may encourage more comprehensive cardiovascular risk assessment and reinforce the importance of aggressive secondary prevention strategies. Beyond the acute setting, these findings also underscore the potential relevance of primary prevention strategies in patients with chronic HCV infection.

Future multicenter prospective studies involving larger HCV-positive STEMI populations are needed to better clarify the relationship between chronic HCV infection, coronary anatomical complexity, inflammatory burden, antiviral therapy, and long-term cardiovascular outcomes. In particular, evaluating the impact of direct-acting antiviral therapy on coronary atherosclerotic progression may provide important mechanistic and therapeutic insights.

## 5. Limitations

Several limitations of this study should be acknowledged. Although the retrospective observational design precludes causal inference, the primary aim was to examine the association between chronic HCV infection and coronary anatomical complexity in a contemporary STEMI population. To reduce confounding, propensity score matching incorporating key clinical and laboratory variables was applied, thereby reducing the impact of measurable confounding.

The relatively low prevalence of HCV infection reflects its frequency in contemporary STEMI cohorts, and the consistency of findings across unmatched and matched analyses argues against substantial selection bias. Restricting the study population to STEMI patients undergoing primary PCI improved cohort homogeneity and interpretability.

Detailed data regarding HCV duration, viral load, genotype, and prior antiviral therapy were unavailable, precluding assessment of potential dose–response relationships and limiting mechanistic interpretation of the observed association. In addition, although SYNTAX scoring was performed in the acute STEMI setting, all angiograms were evaluated by experienced interventional cardiologists blinded to HCV status, thereby minimizing measurement bias.

The absence of long-term follow-up limits prognostic interpretation, as the primary focus of the study was coronary anatomical complexity rather than long-term clinical outcomes. Finally, while the single-center design may limit generalizability, the use of standardized STEMI management protocols and validated angiographic scoring systems supports the external relevance of the findings.

Overall, despite these limitations, this study provides additional evidence supporting an association between chronic HCV infection and increased coronary artery disease complexity in a well-defined STEMI population.

## 6. Conclusions

In patients presenting with ST-segment elevation myocardial infarction, chronic hepatitis C virus infection was independently associated with greater coronary anatomical complexity as assessed by the SYNTAX score, rather than with differences in early in-hospital clinical outcomes. These findings support the concept that HCV infection may contribute to a more diffuse and structurally complex atherosclerotic phenotype beyond traditional cardiovascular risk factors. Recognition of HCV status in patients with STEMI may therefore provide additional insight into underlying coronary disease burden and may help guide cardiovascular risk assessment and secondary prevention strategies. Further prospective multicenter studies are needed to determine whether successful HCV eradication favorably influences coronary disease progression and long-term cardiovascular outcomes.

## Figures and Tables

**Table 1:** Baseline characteristics of patients before and after propensity score matching.

	Before Propensity Score Matching			After Propensity Score Matching		
Variables	HCV (−) (n: 1541)	HCV (+) (n: 106)	*p* Value	HCV (−) (n: 105)	HCV (+) (n: 105)	*p* Value
Male sex, n (%)	870 (56.5%)	67 (63.2%)	0.175	69 (65.7%)	66 (62.9%)	0.666
Age (years)	60.8 ± 12.9	62.5 ± 9.5	0.167	62.0 ± 12.9	62.4 ± 9.4	0.798
BMI (kg/m^2^)	28.2 ± 4.7	27.1 ± 3.7	0.029	28.0 ± 4.4	27.2 ± 3.7	0.166
Systolic BP (mmHg)	133.9 ± 30.7	134.2 ± 26.4	0.938	136.3 ± 33.2	134.0 ± 26.5	0.587
Diastolic BP (mmHg)	77.2 ± 17.5	74.3 ± 14.0	0.094	78.0 ± 19.1	74.3 ± 14.1	0.113
Heart rate (beats/min)	82.9 ± 21.4	81.5 ± 18.3	0.523	82.0 ± 20.4	81.6 ± 18.3	0.876
Hypertension, n (%)	805 (52.2%)	62 (58.5%)	0.212	62 (59.0%)	61 (58.1%)	0.889
Diabetes Mellitus, n (%)	509 (33.0%)	42 (39.6%)	0.164	39 (37.1%)	41 (39.0%)	0.776
Smoking, n (%)	793 (51.5%)	61 (57.5%)	0.225	67 (63.8%)	60 (57.1%)	0.323
Prior Statin Use, n (%)	285 (18.5%)	14 (13.2%)	0.172	20 (19.0%)	14 (13.3%)	0.261
Prior ASA Use, n (%)	396 (25.7%)	19 (17.9%)	0.075	29 (27.6%)	19 (18.1%)	0.100
Atrial Fibrillation, n (%)	73 (4.7%)	15 (14.2%)	<0.001	5 (4.8%)	14 (13.3%)	0.030
LVEF (%)	45.9 ± 10.8	46.2 ± 8.6	0.839	46.5 ± 11.4	46.2 ± 8.7	0.838
Hemoglobin (g/dL)	13.6 ± 2.0	13.3 ± 1.4	0.119	13.4 ± 1.9	13.3 ± 1.4	0.646
Platelet Count (×10^3^/µL)	240.3 ± 69.4	229.9 ± 75.3	0.138	251.2 ± 73.5	230.8 ± 75.1	0.048
WBC (×10^3^/µL)	12.0 ± 3.7	12.3 ± 3.3	0.576	12.0 ± 3.5	12.3 ± 3.3	0.515
Neutrophil (×10^3^/µL)	9.4 ± 3.6	9.5 ± 2.6	0.714	9.4 ± 3.4	9.5 ± 2.6	0.773
Lymphocyte (×10^3^/µL)	1.9 ± 1.1	1.9 ± 0.9	0.897	1.9 ± 1.1	1.9 ± 0.9	0.901
ALT (U/L)	35.2 ± 27.9	40.5 ± 25.3	0.057	35.8 ± 25.0	40.2 ± 25.2	0.211
AST (U/L)	74.9 ± 68.1	67.0 ± 41.9	0.240	75.3 ± 67.5	66.6 ± 41.8	0.263
ALP (U/L)	76.9 ± 31.2	81.2 ± 42.0	0.185	76.3 ± 23.0	81.4 ± 42.2	0.277
GGT (U/L)	37.5 ± 35.4	44.0 ± 25.4	0.065	42.7 ± 46.6	44.0 ± 25.6	0.807
Total Bilirubin (mg/dL)	1.3 ± 4.6	1.4 ± 1.9	0.755	1.8 ± 7.2	1.4 ± 1.9	0.607
Uric Acid (mg/dL)	6.2 ± 2.0	6.3 ± 1.6	0.827	6.2 ± 2.0	6.2 ± 1.5	0.849
Serum Albumin (g/dL)	3.8 ± 0.4	3.7 ± 0.5	0.022	3.8 ± 0.3	3.7 ± 0.5	0.038
CRP (mg/L)	2.7 ± 3.8	4.0 ± 3.0	<0.001	3.1 ± 4.1	4.0 ± 3.0	0.071
INR	1.2 ± 0.2	1.2 ± 0.4	0.263	1.2 ± 0.2	1.2 ± 0.4	0.389
Total Cholesterol (mg/dL)	198.1 ± 47.4	179.4 ± 54.4	<0.001	194.3 ± 37.3	179.8 ± 54.5	0.028
LDL Cholesterol (mg/dL)	116.2 ± 38.6	98.9 ± 45.3	<0.001	122.3 ± 31.4	99.2 ± 45.4	<0.001
HDL Cholesterol (mg/dL)	37.2 ± 9.0	36.3 ± 11.4	0.365	38.0 ± 8.9	36.4 ± 11.5	0.243
Triglycerides (mg/dL)	215.2 ± 192.8	210.4 ± 165.8	0.774	211.9 ± 193.6	209.9 ± 165.9	0.689
Anti-HCV (S/CO)	N/A	39.4 ± 52.2	-	N/A	39.5 ± 51.9	-
Revascularization strategy PCI, n (%) CABG, n (%)	1459 (94.7%) 82 (5.3%)	94 (88.7%) 12 (11.3%)	0.010	102 (97.1%) 3 (2.9%)	93 (88.6%) 12 (11.4%)	0.016
SYNTAX Score	15.8 ± 9.6	19.5 ± 8.5	<0.001	15.6 ± 9.2	19.4 ± 8.5	0.002
In-Hospital Mortality, n (%)	157 (10.2%)	11 (10.4%)	0.950	9 (8.6%)	11 (10.5%)	0.638

Abbreviations: ALP, alkaline phosphatase; ALT, alanine aminotransferase; ASA, acetylsalicylic acid; AST, aspartate aminotransferase; BMI, body mass index; BP, blood pressure; CABG, coronary artery bypass grafting; CRP, C-reactive protein; GGT, gamma-glutamyl transferase; HCV, hepatitis C virus; HDL, high-density lipoprotein; INR, international normalized ratio; LDL, low-density lipoprotein; LVEF, left ventricular ejection fraction; PCI, percutaneous coronary intervention; WBC, white blood cell count.

**Table 2:** Conventional linear regression analysis for SYNTAX score before propensity score matching.

	Univariable		Multivariable	
Variables	Estimate (CI %95)	*p*-Value	Estimate (CI %95)	*p*-Value
Age (years)	0.075 (0.039, 0.112)	<0.001	0.066 (0.025, 0.106)	0.001
Diabetes mellitus (yes/no)	3.160 (2.180, 4.130)	<0.001	2.616 (1.604, 3.602)	<0.001
Hypertension (yes/no)	2.720 (1.800, 3.640)	<0.001	1.695 (0.706, 2.685)	<0.001
Smoking (yes/no)	0.155 (−0.773, 1.080)	0.740	1.670 (0.656, 2.684)	0.001
Neutrophil (×10^3^/µL)	0.270 (0.140, 0.395)	<0.001	0.244 (0.117, 0.371)	<0.001
CRP (mg/L)	−0.003 (−0.129, 0.121)	0.952	−0.064 (−0.188, 0.059)	0.309
Serum Albumin (g/dL)	−1.500 (−2.200, −0.079)	0.035	−0.998 (−2.068, 0.069)	0.067
HCV (yes/no)	3.680 (1.800, 5.561)	<0.001	3.128 (1.280, 4.979)	<0.001

Abbreviations: CI, Confidence Interval; CRP, C-reactive protein; HCV, hepatitis C virus.

**Table 3:** Linear regression analysis for SYNTAX score after propensity score matching.

	Univariable		Multivariable	
Variables	Estimate (CI %95)	*p*-Value	Estimate (CI %95)	*p*-Value
Age (years)	0.082 (−0.026, 0.191)	0.136	0.025 (−0.086, 0.138)	0.652
Diabetes mellitus (yes/no)	6.30 (3.92, 8.68)	<0.001	6.36 (3.877, 8.852)	<0.001
Hypertension (yes/no)	1.95 (−0.530, 4.43)	0.123	1.021 (−1.495, 3.538)	0.425
Smoking (yes/no)	0.01 (−2.51, 2.53)	0.990	2.12 (−0.480, 4.720)	0.109
Neutrophil (×10^3^/µL)	0.001 (−0.404, 0.406)	0.990	−0.177 (−0.570, 0.215)	0.374
CRP (mg/L)	0.038 (−0.302, 0.379)	0.820	0.013 (−0.310, 0.340)	0.933
Serum Albumin (g/dL)	−0.84 (−3.63, 1.94)	0.550	0.154 (−2.593, 2.902)	0.912
HCV (yes/no)	3.76 (1.35, 6.16)	0.003	3.805 (1.472, 6.139)	0.002

Abbreviations: CI, Confidence Interval; CRP, C-reactive protein; HCV, hepatitis C virus.

**Table 4:** Logistic regression analysis for in-hospital mortality.

Variables	Odds Ratio (CI %95)	*p* Value
Age	1.055 (1.041, 1.070)	<0.001
HT (yes/no)	0.881 (0.610, 1.275)	0.503
DM (yes/no)	1.964 (1.386, 2.785)	<0.001
SYNTAX Score	1.001 (0.985, 1.018)	0.950
Ejection Fraction (%)	0.992 (0.978, 1.008)	0.370
Hemoglobin	1.042 (0.956, 1.137)	0.346
WBC	0.972 (0.929, 1.018)	0.243
HCV (yes/no)	0.998 (0.513, 1.940)	0.978

Abbreviations: CI, Confidence Interval; DM, diabetes mellitus; HCV, hepatitis C virus; HT, hypertension; WBC, white blood cell count.

## Data Availability

The data underlying this article will be shared by the corresponding author upon reasonable request.

## Supplementary Materials

The following supporting information can be downloaded at: https://www.mdpi.com/article/10.3390/jcm15114180/s1, Figure S1: Covariate balance before and after propensity score matching (Love plot); Figure S2: Example application of the nomogram for individualized prediction of SYNTAX score.
