# Supplementary material for: Association Between Hepatitis C Virus Infection and SYNTAX Score in Patients with ST-Segment Elevation Myocardial Infarction: A Propensity Score-Matched Analysis
Source: J Clin Med. 2026 May 28;15(11):4180. doi: 10.3390/jcm15114180 (PMC13257680; doi:10.3390/jcm15114180)
Supplement: Supplementary file 1 [file jcm-15-04180-s001.zip › jcm-4315080-supplementary.pdf]

Supplementary Figure S1. Covariate balance before and after propensity score matching (Love plot).

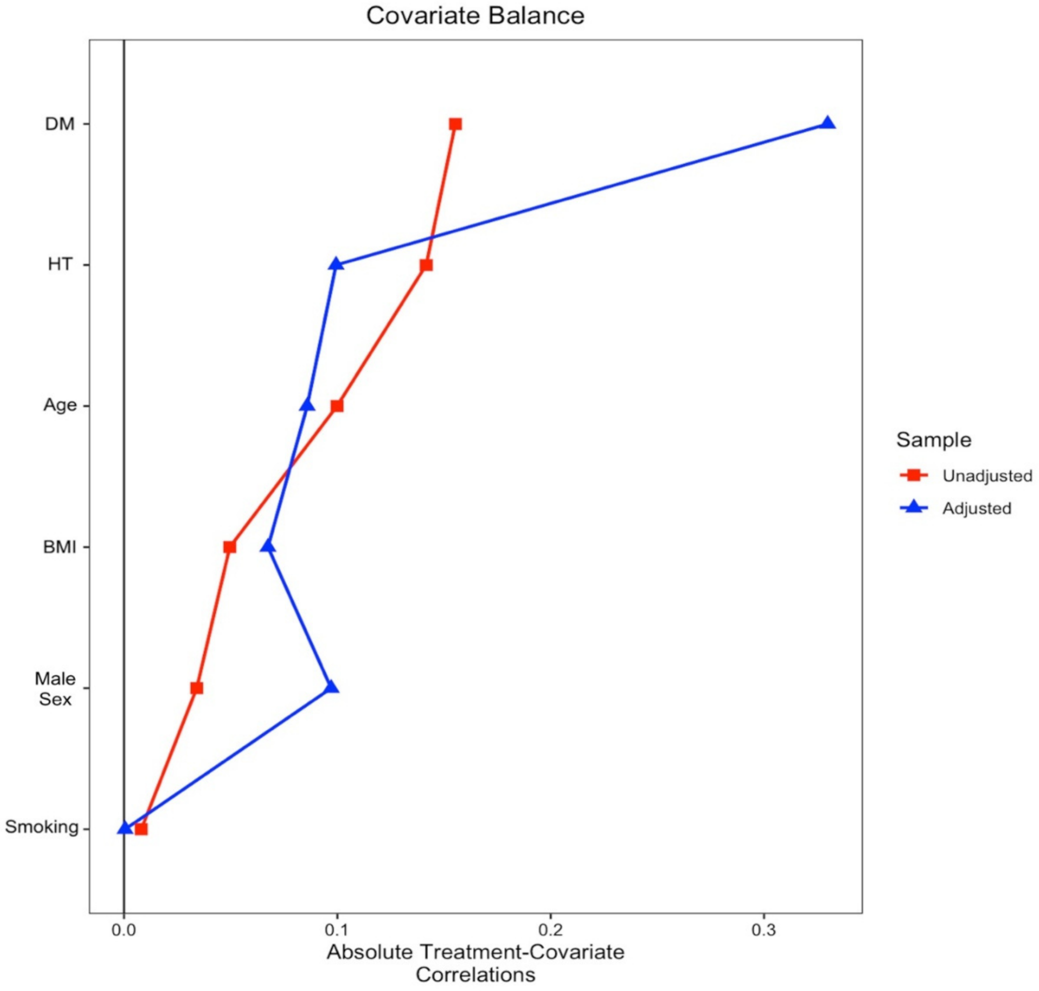

Supplementary Figure S2. Example application of the nomogram for individualized prediction of SYNTAX score.

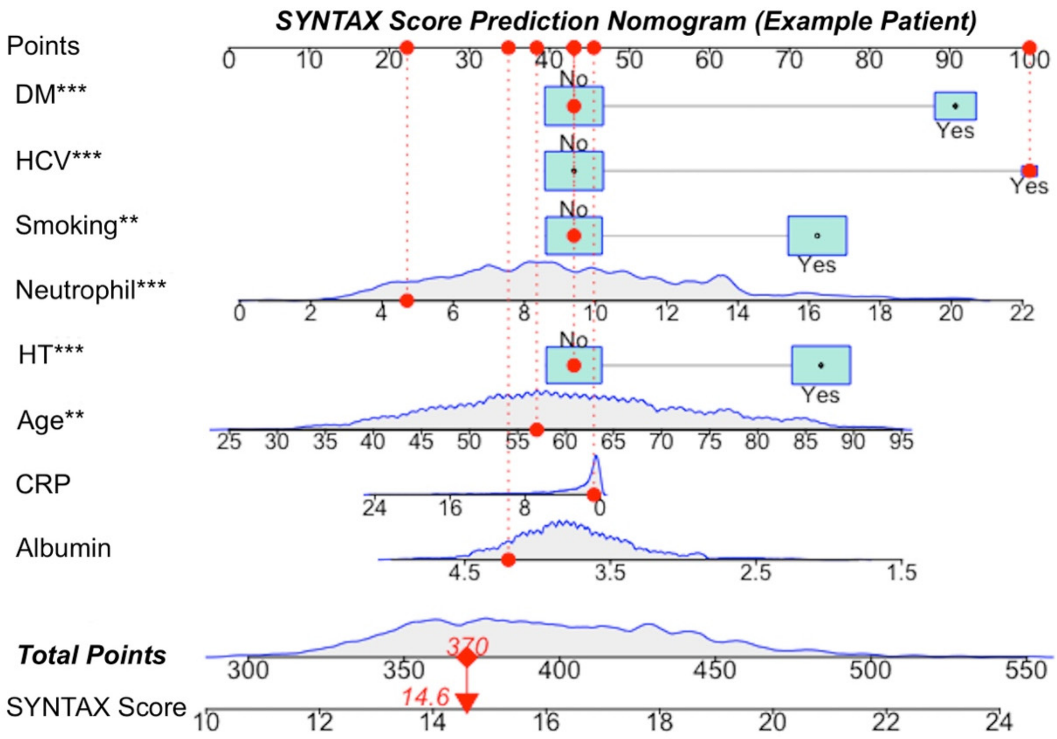

Illustrative example demonstrating the application of the nomogram in an individual patient, showing the calculation of the total point score and the corresponding estimated SYNTAX score.
